# Descriptive Epidemiology and Prognostic Significance of Diaphragm Thickness in the General Population: The Nagahama Study

**DOI:** 10.1002/jcsm.13690

**Published:** 2025-01-25

**Authors:** Yasuharu Tabara, Takeshi Matsumoto, Kimihiko Murase, Takahisa Kawaguchi, Kazuya Setoh, Tomoko Wakamura, Toyohiro Hirai, Kazuo Chin, Fumihiko Matsuda

**Affiliations:** ^1^ Graduate School of Public Health Shizuoka Graduate University of Public Health Shizuoka Japan; ^2^ Center for Genomic Medicine Kyoto University Graduate School of Medicine Kyoto Japan; ^3^ Department of Respiratory Medicine Kyoto University Graduate School of Medicine Kyoto Japan; ^4^ Department of Human Health Science Kyoto University Graduate School of Medicine Kyoto Japan; ^5^ Department of Sleep Medicine and Respiratory Care, Division of Sleep Medicine Nihon University of Medicine Tokyo Japan

**Keywords:** all‐cause mortality, diaphragm thickness, pulmonary function, sarcopenia

## Abstract

**Background:**

Diaphragm thickness is a potential marker of sarcopenia in addition to muscle mass and strength at extremities. We aimed to clarify the descriptive epidemiology and prognostic significance of diaphragm thickness in the general population.

**Methods:**

The study participants were 3324 community residents (mean age: 61.4 ± 12.8 years) who participated in a longitudinal cohort study. Clinical parameters were obtained during the follow‐up survey of the study population. Diaphragm thickness was measured from B‐mode ultrasound images obtained in a supine position. Clinical and physical factors independently associated with diaphragm thickness were assessed by a linear regression model and a causal mediation analysis. All‐cause mortality was determined by reviewing residential registry records. Prognostic significance of diaphragm thickness for all‐cause mortality was examined using a Cox proportional hazard model analysis.

**Results:**

Diaphragm thickness was greater in men than women (end‐expiration, β = 0.161, *p* < 0.001; end‐inspiration, β = 0.156, *p* < 0.001) and associated with waist circumference (end‐expiration, β = 0.259, *p* < 0.001; end‐inspiration, β = 0.128, *p* < 0.001). Handgrip strength, smoking habit, insulin resistance and exercise habit were not associated with diaphragm thickness. Skeletal muscle mass index showed apparent association with diaphragm thickness, though this association was not observed after adjusting for waist circumference. Over a mean follow‐up of 1686 days (15 358 person‐years), there were 56 cases of all‐cause mortality. Weak handgrip strength (hazard ratio = 0.95, *p* = 0.044) and low forced vital capacity (hazard ratio = 0.57, *p* = 0.045) were associated with all‐cause mortality, though none of the diaphragm thickness parameters showed a significant association (thickness at end‐expiration, *p* = 0.722; thickness at end‐inspiration, *p* = 0.277; thickening fraction, *p* = 0.219).

**Conclusions:**

Waist circumference but not parameters of sarcopenia was independently associated with diaphragm thickness. Diaphragm thickness was not associated with all‐cause mortality. Diaphragm thickness may not be a marker of systemic sarcopenia.

## 1 Introduction

Sarcopenia in older adults is a risk factor for age‐related morbidity such as falls and fractures [1], loss of physical independence [2] and cognitive impairment [3]. Studies have also found an association between sarcopenia and all‐cause mortality [4, 5]. Sarcopenia is a composite phenotype including both a decrease in muscle mass and an attenuation of muscle function. The European [6] and Asian [7] working groups on sarcopenia advocate that the diagnosis of sarcopenia should be based on both low muscle mass and weak muscle strength or reduced physical performance. Among these parameters required for the diagnosis of sarcopenia, muscle mass is usually assessed by skeletal muscle mass index (SMI), which is calculated by subdividing appendicular lean mass estimated from the bioelectrical impedance measures by squared‐body height [6, 7]. Bioelectrical impedance analysis is a convenient and non‐invasive method for the assessment of muscle mass and is widely used in epidemiological studies and community health practice.

Age‐related deterioration of skeletal muscles in extremities can occur concomitantly with the deterioration of muscles in other parts of the body. The diaphragm is a skeletal muscle that plays a pivotal role in respiration and can be assessed non‐invasively by ultrasonography [8]. Reduced pulmonary function was shown to be associated with case‐specific [9, 10] and all‐cause mortality [10, 11, 12] independent of muscle strength [13]. Given these previous findings, physiological changes in the diaphragm may partly be involved in the association between reduced pulmonary function and mortality and may be used as another marker of systemic sarcopenia that cannot be assessed by the measurement of skeletal muscle properties in extremities alone. Indeed, a previous study [14] found reduced thickness of the diaphragm in older adults with sarcopenia, although the study had a small sample size. Another cross‐sectional study in older adults [15] reported positive correlations between diaphragm thickness and handgrip strength, appendicular lean mass, calf circumference and gait speed, though the results of this simple correlation analysis alone did not support the involvement of diaphragm thickness due to insufficient adjustment of possible confounding factors. Furthermore, there is a paucity of studies on the epidemiology and prognostic significance of diaphragm thickness in the general population.

The aim of this study was to identify factors associated with diaphragm thickness and to clarify the prognostic significance of diaphragm thickness in a large population‐based longitudinal cohort study.

## 2 Methods

### 2.1 Study Participants

We analysed a dataset from the Nagahama study, a longitudinal cohort study of community residents in Nagahama City, a suburban city located in central Japan [16, 17]. The study participants were recruited between 2008 and 2010 among community residents aged 30–74 years who were living without any physical assistance. In the Nagahama study, a clinical survey was performed every 5 years after the baseline survey. The present study analysed data obtained in the former half (2017 and 2018) of the third (second follow‐up) survey. Out of a total of 3443 participants, 3324 were included in the analysis after applying the following exclusion criteria: pregnant women (*N* = 3), unmeasurable diaphragm echography (*N* = 3) or spirometry (*N* = 61) and clinical values deviating significantly from the distribution (*N* = 52).

All study procedures of the Nagahama study were approved by the ethics committee of the Kyoto University Graduate School of Medicine and the Nagahama Municipal Review Board. Written informed consent was obtained from all participants prior to enrolment.

### 2.2 Death Ascertainment

All‐cause mortality was identified by reviewing residential registry records managed by the Nagahama City Office. Participants who were relocated out of Nagahama City were censored. The follow‐up period was calculated from the second follow‐up survey to the date of relocation or death or the current end of the follow‐up period (31 March 2023).

### 2.3 Diaphragm Ultrasonography

Diaphragm thickness was measured from B‐mode ultrasound images obtained under maximal effort. The images were obtained in a supine position using UF‐760AG (Fukuda Denshi Co. Ltd., Tokyo, Japan) with a 7.5‐MHz linear probe. The measurements were performed by well‐trained sonographers in a predefined manner, that is, the transducer was placed over the eighth or ninth right intercostal space in the midaxillary or anterior axillary line [8], and two different images were obtained at end‐expiration and end‐inspiration (four images in total). Diaphragm thickness was measured as the distance between the pleural and peritoneal membranes [8]. Measurement of the thickness was taken at one point on each image, and mean thickness at end‐expiration and end‐inspiration was used for the analysis. Intraclass correlation coefficient calculated as a composite measure of intra‐observer and inter‐day variability in the measurement of diaphragm thickness was 0.694 for the thickness at end‐expiration and 0.757 for the thickness at end‐inspiration. The thickening fraction was calculated as a percent change in the diaphragm thickness during inspiration using the following formula: [(thickness at end‐inspiration − thickness at end‐expiration)/thickness at end‐expiration] × 100.

### 2.4 Measurement of Body Composition

Fat mass and appendicular lean mass was estimated using a bioelectrical impedance analysis device (InBody 430; InBody Co. Ltd., Seoul, Korea). This device can estimate fat mass and lean mass from the resistance and reactance of arms, the trunk and legs at three different frequencies (5, 50 and 250 kHz) of an alternating 250 A current. SMI was calculated by dividing the appendicular lean mass by the squared‐body height in meters [7]. Studies have demonstrated the validity and reproducibility of lean mass measurement by this segmental multiple‐frequency bioelectrical impedance analysis compared to that measured by dual‐energy x‐ray absorptiometry [18, 19] and hydrostatic weighing [20].

### 2.5 Measurement of Handgrip Strength

The handgrip strength of the dominant hand was measured using a digital grip dynamometer (Grip‐D; Takei Scientific Instrument Co. Ltd., Niigata, Japan). The measurement was made with the participant in the sitting position and the arm placed on the desk (70 cm in height), horizontal to the ground to avoid postural effect on grip strength [21]. Participants were instructed to adjust the handle of the dynamometer to be under the second phalanx of the fingers when gripped. Two measurements were obtained for each participant with a few second interval, and the larger of the two values was used for the analysis. If the subject was unable to use the dominant hand, measurements were taken with the non‐dominant hand.

### 2.6 Assessment of Pulmonary Function

Pulmonary function was measured by forced vital capacity (FVC) manoeuvre using a computed spirometer with automated quality checks (Spiro Sift SP370HYPER, Fukuda Denshi, Tokyo, Japan). Prebronchodilator spirometry was performed by certified medical technologists following a standardized protocol [22]. Measurements were obtained twice, and the best measurement was used for the analysis. FVC, forced expiratory volume in 1 s (FEV_1_) and the FEV_1_/FVC ratio were used as pulmonary function indices. History of pulmonary diseases including bronchial asthma and chronic obstructive pulmonary disease that are frequently observed in a general population was queried using a structure questionnaire. Because our study participants were community‐dwelling apparently healthy individuals, lung cancer was not considered in the analysis. The use of inhaled medication was also observed using the questionnaire.

### 2.7 Measurement of Advanced Glycation End Products (AGEs)

The accumulation of advanced glycation end products (AGEs) was measured using the skin autofluorescence (SAF) technique. In brief, SAF‐AGEs were measured on the middle finger of the non‐dominant hand using a prototype of the AGE sensor RQ‐AG01J (Air Water Biodesign Inc., Kobe, Japan). The fluorescence emission spectrum (440–460 nm) excited by a light‐emitting diode (365 nm) was measured on a skin surface area approximately 0.38 mm in diameter using a 2048‐pixel charge‐coupled device linear image sensor [23]. Measurements were taken in triplicate, and the mean value was calculated for the analysis. The coefficient of variation and intraclass correlation coefficient of the repeated measurements were 6.65 ± 7.25% and 0.938, respectively. The accuracy of this non‐invasive method has been verified by comparing SAF‐AGE levels to the tissue levels of major AGE molecules, that is, carboxymethyl lysine and pentosidine [24].

### 2.8 Basic Clinical Parameters

Basic clinical pwarameters analysed in this study were obtained at the second follow‐up survey of the Nagahama study. Plasma levels of glycaemic markers including glucose, insulin and haemoglobin A1c were considered in the analysis because hyperglycaemia has been reported to be a risk factor for sarcopenia [25]. Homeostasis model assessment of insulin resistance (HOMA‐IR) was used as an index of insulin resistance and calculated using the formula: plasma glucose × insulin/405. Menopausal status, smoking and exercise habits, disease history and medication use were queried using a structured questionnaire. Exercise habit was assessed by the following yes‐no question: ‘Do you engage in moderate‐intensity exercise (e.g., sweat lightly) at least twice a week for 30 min over a period of 1year?’ [26]. This question is included in a questionnaire used in the nationwide health check‐up system in Japan.

### 2.9 Statistical Analysis

Data are presented as mean ± standard deviation or frequency (percentage). Quartiles of the numeric variables were calculated by sex and combined to avoid potential sex differences. Inter‐group differences with respect to continuous variables were assessed by analysis of variance. Trend analysis was performed using the Jonckheere–Terpstra test. Linear regression analysis was performed to identify factors independently associated with diaphragm thickness and pulmonary function. Causal mediation analysis was used to assess the confounding influence of body weight in the association between appendicular lean mass and diaphragm thickness. The Cox proportional hazard model was used to identify factors associated with all‐cause mortality.

Statistical analyses were performed using the JMP Pro 17.2.0 software (SAS Institute, NC, USA). The STATA 18.0 software (Stata Corp LLC, Texas, USA) was used for Jonckheere–Terpstra test and causal mediation analysis. *p* values less than 0.05 were considered indicative of statistical significance.

## 3 Results

The clinical characteristics of the study participants are summarized in Table 1. The mean age of the total study population was 61.4 ± 12.8 years, whereas age distribution in this population is shown in Figure S1.

**TABLE 1:** Clinical characteristics of the study participants.

	Men	Women
	*N* = 1006	*N* = 2318
Age, years	63.6 ± 13.2	60.4 ± 12.5
Smoking, current/past/never %	18.3/49.5/32.2	3.0/9.1/87.9
Exercise habit, %	35.8	31.1
SAF‐AGEs, arbitrary unit	1550 ± 481	1472 ± 482
Physical parameters		
Body height, cm	167.8 ± 6.5	155.7 ± 6.2
Body weight, kg	65.8 ± 10.2	53.2 ± 8.8
BMI, kg/m^2^	23.3 ± 3.0	22.0 ± 3.4
Waist circumference, cm	83.9 ± 8.4	79.5 ± 9.2
Fat mass, kg	14.6 ± 5.8	15.5 ± 6.2
SMI, kg/m^2^	7.7 ± 0.7	6.2 ± 0.7
Handgrip strength, kg	36.6 ± 7.7	23.1 ± 5.0
Plasma markers		
Glucose, mg/dL	93 ± 16	86 ± 11
Insulin, μU/mL	4.4 ± 4.6	3.6 ± 2.9
HOMA‐IR	1.06 ± 1.44	0.80 ± 0.82
HbA1c, %	5.6 ± 0.5	5.6 ± 0.4
Pulmonary parameters		
FVC, L	3.8 ± 0.8	2.8 ± 0.5
FEV_1_, L	2.9 ± 0.7	2.2 ± 0.5
FEV_1_/FVC, %	75.8 ± 7.4	78.4 ± 5.8
Inhaled medication, %	1.6	1.7
Pulmonary disease, %	5.8	5.0

*Note:* Data presented as mean ± standard deviation or frequency. Pulmonary diseases include bronchial asthma and chronic obstructive pulmonary disease. As shown in the Results, fat mass is used to various statistical analysis (highlighted in red).

Abbreviations: BMI, body mass index; FEV_1_, forced expiratory volume in 1 s; FVC, forced vital capacity; HbA1c, haemoglobin A1c; HOMA‐IR, homeostasis model assessment of insulin resistance; SAF‐AGEs, skin autofluorescence of advanced glycation end products; SMI, skeletal muscle mass index.

**FIGURE 1:**
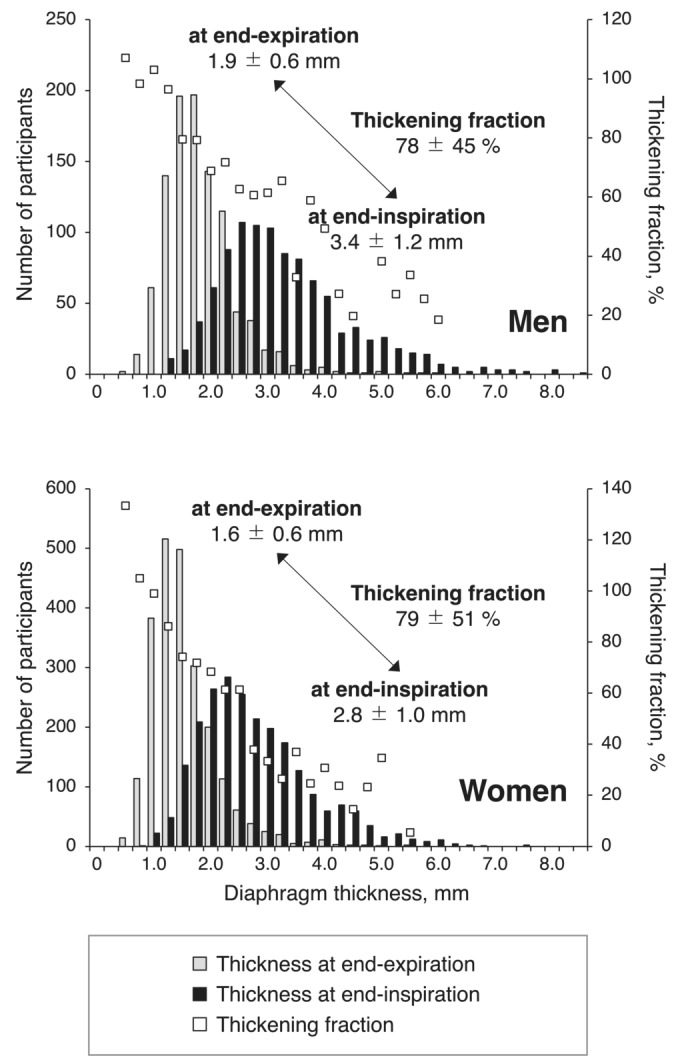
Distribution of diaphragm thickness and thickening fraction. The mean thickening fraction was calculated in each subgroup of the diaphragm thickness at end‐expiration.

The distribution of diaphragm thickness at end‐expiration and end‐inspiration is depicted in Figure 1. The diaphragm thickness at end‐expiration and end‐inspiration was significantly greater in men (*p* < 0.001 for both), although there were no significant differences between men and women in terms of the thickening fraction (*p* = 0.737). A linear inverse correlation was observed between diaphragm thickness at end‐expiration and the thickening fraction (men, *r* = −0.321, *p* < 0.001; women, *r* = −0.268, *p* < 0.001) (Figure 1), with the thinner the diaphragm at end‐expiration, the greater the thickening fraction.

**FIGURE 2:**
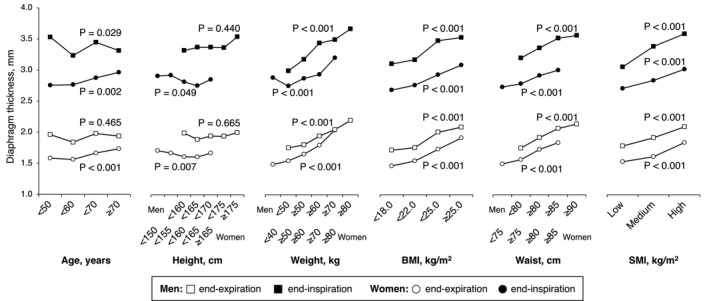
Association of diaphragm thickness with age and anthropometric factors. Data presented as mean. Statistical significance was assessed using the Jonckheere–Terpstra test. BMI, body mass index; SMI, skeletal muscle mass index; Waist, waist circumference.

Figure 2 shows the association of age and anthropometric factors with diaphragm thickness in a trend analysis by the Jonckheere–Terpstra test. Simple correlation coefficients among these factors are summarized in Table S1. In both analyses, body weight but not body height showed a positive association with diaphragm thickness, indicating that the positive association between body mass index and diaphragm thickness was attributed to the positive association with body weight. Waist circumference was another factor that was strongly associated with diaphragm thickness (Figure 2 and Table S2). When both body weight and waist circumference were included in a same linear regression model, waist circumference showed superior association with diaphragm thickness at end‐expiration (men: waist circumference, *p* < 0.001, body weight, *p* = 0.818; women: waist circumference, *p* < 0.001, body weight, *p* = 0.164) and at end‐inspiration in women (waist circumference, *p* = 0.001, body weight = 0.804), though clear superiority was not observed in men (waist circumference, *p* = 0.503, body weight, *p* = 0.125). A superior associations of waist circumference with diaphragm thickness at end‐expiration (men: waist circumference, *p* < 0.001, fat mass, *p* = 0.172; women: waist circumference, *p* < 0.001, fat mass, *p* = 0.075) and at end‐inspiration (men: waist circumference, *p* = 0.006, fat mass, *p* = 0.204; women: waist circumference, *p* = 0.003, fat mass, *p* = 0.956) were also observed in the analysis including fat mass instead of body weight in a regression model. Given these results, in the following analysis, waist circumference was considered as an anthropometric factor associated with diaphragm thickness. The association between waist circumference and diaphragm thickness remained significant in the analysis further adjusted for potential covariates including age, sex, smoking habit, insulin resistance, handgrip strength and exercise habit (Table 2). In this analysis, handgrip strength (a representative measure of systemic muscle strength), insulin resistance (a potential risk factor for loss of muscle mass) and exercise habit were not identified as significant determinants even in the sex separated analysis (Table S2). In addition to waist circumference, SAF‐AGEs showed an inverse association with diaphragm thickness (Table 3).

**TABLE 2:** Linear regression analysis for diaphragm thickness.

	Diaphragm thickness				Diaphragm thickening fraction	
	At end‐expiration		At end‐inspiration			
	β	*p*	β	*p*	β	*p*
Age, years	0.019	0.340	0.018	0.376	< 0.000	0.987
Sex, men	0.161	< 0.001	0.156	< 0.001	−0.007	0.811
Waist circumference, cm	0.259	< 0.001	0.128	< 0.001	−0.152	< 0.001
Smoking habit	−0.013	0.529	0.001	0.954	0.011	0.599
Handgrip strength, kg	0.012	0.652	0.063	0.019	0.044	0.109
HOMA‐IR, log normalized	−0.030	0.118	−0.037	0.059	−0.026	0.203
Exercise habit	0.051	0.003	0.030	0.087	−0.028	0.126

*Note:* Smoking habit includes current and past smokers.

Abbreviations: HOMA‐IR, homeostasis model assessment of insulin resistance; VIF, variance inflation factor; β, standardized regression coefficient.

**TABLE 3:** Differences in diaphragm thickness among the quartiles of SAF‐AGEs.

			Diaphragm thickness				Diaphragm thickening fraction	
			At end‐expiration		At end‐inspiration			
			Mean ± SD	*p*	Mean ± SD	*p*	Mean ± SD	*p*
SAF‐AGEs	Q1	(830)	1.79 ± 0.65	Reference	3.09 ± 1.14	Reference	77.0 ± 46.8	
	Q2	(832)	1.76 ± 0.65	0.824	3.05 ± 1.09	0.646	78.7 ± 48.2	0.847
	Q3	(832)	1.74 ± 0.63	0.390	3.02 ± 1.03	0.314	79.3 ± 49.8	0.567
	Q4	(830)	1.64 ± 0.53	< 0.001	2.88 ± 1.00	< 0.001	79.7 ± 51.8	0.620

*Note:* Quartiles of skin autofluorescence of advanced glycation end products (SAF‐AGEs) were calculated separately for men and women and then combined to avoid potential sex‐based differences. The number of participants in each group is shown in parentheses. Statistical significance was assessed by linear regression analysis adjusted for age, sex, waist circumference, smoking habit, handgrip strength, homeostasis model assessment of insulin resistance (log normalized) and exercise habit.

SMI also showed a positive association with diaphragm thickness (Figure 2). The strong positive correlation between waist circumference and SMI (men, *r* = 0.547, *p* < 0.001; women, *r* = 0.515, *p* < 0.001) indicated a potential confounding influence of waist circumference in the association between SMI and diaphragm thickness. To clarify this possibility, a causal mediation analysis using waist circumference as a mediation factor was performed (Table 4 and Figure S2). In this analysis, appendicular lean mass but not SMI was included in the model because of the lack of association between body height and diaphragm thickness. The results showed no direct association between appendicular lean mass and diaphragm thickness, but the indirect effect via waist circumferences was statistically significant, suggesting that appendicular lean mass was not an independent determinant of diaphragm thickness. Similar results were observed in the analysis of older (≥ 65 years of age, *n* = 1625) subpopulation (Table S3). Simple linear regression analysis adjusted for age and sex also showed superior association of waist circumference than appendicular lean mass with both diaphragm thickness at end‐expiration (waist circumference, β = 0.216, *p* < 0.001; appendicular lean mass, β = 0.079, *p* = 0.016) and end‐inspiration (waist circumference, β = 0.086, *p* < 0.001; appendicular lean mass β = 0.080, *p* = 0.017).

**TABLE 4:** Causal mediation analysis between appendicular lean mass and diaphragm thickness.

	Appendicular lean mass	Diaphragm thickness					
		At end‐expiration			At end‐inspiration		
		Coefficient	SE	*p*	Coefficient	SE	*p*
Natural indirect effect via waist circumference	Q1		Reference			Reference	
	Q2	0.031	0.009	< 0.001	0.027	0.010	0.011
	Q3	0.069	0.012	< 0.001	0.066	0.018	< 0.001
	Q4	0.141	0.020	< 0.001	0.111	0.033	0.001
Natural direct effect	Q1						
	Q2	−0.001	0.028	0.982	0.024	0.051	0.633
	Q3	−0.011	0.030	0.714	0.019	0.056	0.730
	Q4	0.055	0.037	0.139	0.063	0.065	0.329
Total effect	Q1						
	Q2	0.031	0.029	0.289	0.051	0.051	0.316
	Q3	0.058	0.031	0.059	0.085	0.055	0.122
	Q4	0.196	0.036	< 0.001	0.175	0.060	0.004

*Note:* Adjusted factors were age, sex, smoking habit, homeostasis model assessment of insulin resistance (log normalized), grip strength and exercise habit. Quartiles of appendicular lean mass were calculated separately for men and women and then combined to avoid potential sex‐based differences. SE indicates standardized error.

The results of the linear regression analysis for spirometry parameters are summarized in Table 5, whereas the detailed results are presented in Table S4. Diaphragm thickness at end‐inspiration but not at end‐expiration showed a significant association with FVC and FEV_1_. The thickening fraction also showed a positive association with FVC and FEV_1_, though none of the diaphragm parameters were associated with FEV_1_/FVC.

**TABLE 5:** Linear regression analysis for spirometry parameters.

		FVC		FEV_1_		FEV_1_/FVC	
		β	*p*	β	*p*	β	*p*
Model 1	Diaphragm thickness at end‐expiration, cm	−0.010	0.343	−0.004	0.715	0.017	0.313
Model 2	Diaphragm thickness at end‐inspiration, cm	0.032	0.002	0.032	0.002	0.007	0.697
Model 3	Diaphragm thickening fraction, %	0.042	< 0.001	0.038	< 0.001	−0.007	0.663

*Note:* Analysis was performed in a population not taking inhaled medication and with no history of pulmonary disease (*N* = 3135). Adjusted factors in each model were age, sex, body height, body weight and smoking habit. Detailed results of the regression analysis are shown in Table S3.

Abbreviations: FEV_1_, forced expiratory volume in 1 s; FVC, forced vital capacity; β, standardized regression coefficient.

Over a mean follow‐up duration of 1686 days (15 358 person‐years), there were 56 cases of all‐cause mortality. The Cox proportional hazard model analysis for all‐cause mortality is summarized in Table 6. Handgrip strength but not SMI showed an inverse association with all‐cause mortality after adjusting for age [hazard ratio (1 year) = 1.08, *p* < 0.001], sex [hazard ratio (men) = 3.61, *p* = 0.001], body mass index (*p* = 0.543) and current smoking (*p* = 0.162). FVC also showed an inverse association with all‐cause mortality. However, none of the diaphragm thickness parameters showed a significant association even in the older subpopulation (age ≥ 65 years) (diaphragm thickness at end‐expiration, *p* = 0.812; diaphragm thickness at end‐inspiration, *p* = 0.207; and thickening fraction, *p* = 0.106). In this Cox proportional hazard model, body mass index was included in the model because of its importance as a factor in all‐cause mortality, whereas other regression models for diaphragm thickness included waist circumference. However, when waist circumference was included in the model instead of body mass index, parameters of diaphragm thickness did not show significant association with all‐cause mortality (at end‐expiration, *p* = 0.576; at end‐inspiration, *p* = 0.231; thickening fraction, *p* = 0.219).

**TABLE 6:** Cox proportional hazard model for all‐cause mortality.

	Handgrip strength (kg)	SMI (kg/m^2^)	FVC (L)	Diaphragm thickness		
				At end‐expiration (mm)	At end‐inspiration (mm)	Thickening fraction (%)
Hazard ratio (95% CI)	0.95 (0.91–0.99)	0.71 (0.41–1.26)	0.57 (0.32–0.98)	0.93 (0.59–1.38)	0.87 (0.66–1.11)	1.00 (0.99–1.00)
*p*	0.044	0.246	0.045	0.722	0.277	0.219

*Note:* Adjusted factors were age, sex, body mass index and current smoking. Each factor was included in the Cox hazard model individually.

Abbreviations: FVC, forced vital capacity; SMI, skeletal muscle mass index.

## 4 Discussion

In this large population‐based longitudinal study, increased waist circumference and accumulation of AGEs but not appendicular lean mass were independently associated with diaphragm thickness. Diaphragm thickness showed an independent association with pulmonary function but not with all‐cause mortality.

Contrary to our prior hypothesis, diaphragm thickness was not associated with all‐cause mortality. Despite the small number of deaths during the follow‐up period, weak handgrip strength and low FVC were identified as determinants in this study setting, suggesting very limited prognostic significance of diaphragm thickness, if any, compared to these conventional risk factors. We were not able to provide a cut‐off value for diaphragm thickness to predict all‐cause mortality. Diaphragm thickness did not change with age and was not associated with indices of systemic sarcopenia even in the older subpopulation, suggesting that diaphragm thickness is maintained despite ageing and deteriorating systemic sarcopenia. A thinner diaphragm is currently believed to be a marker of sarcopenia [14]. If so, the lack of association with age and systemic sarcopenia is liable to counteract the association with all‐cause mortality. In older adults, being underweight rather than overweight or obese was reported to be a strong risk factor for all‐cause mortality [27]. The strong positive correlation between diaphragm thickness and waist circumference, as well as body weight, may also have obscured the association between diaphragm thickness and all‐cause mortality. A plausible reason for the positive association between waist circumference and diaphragm thickness may be increases in abdominal fat that shift the diaphragm downwardly, resulting in reduced diaphragm muscle tone and increased diaphragm muscle thickness [28].

Recently, Japanese societies related to respiratory and geriatric medicine proposed the concept of respiratory sarcopenia, a condition characterized by both low respiratory muscle strength and low respiratory muscle mass [29]. Briefly, respiratory sarcopenia was defined by the combination of low respiratory muscle strength (assessed by maximum inspiratory pressure and/or maximal expiratory pressure) and low respiratory muscle mass; diaphragm thickness is an objective measure of the low respiratory muscle mass. It was reported that respiratory sarcopenia assessed by the combination of FVC and appendicular skeletal muscle mass was independently associated with all‐cause mortality in older community‐dwelling residents [30]. A significant association was also observed even when respiratory sarcopenia was assessed using peak expiratory flow rate alone, an alternative measure of respiratory muscle strength [30]. Given the lack of association between diaphragm thickness and all‐cause mortality in the present study, low respiratory muscle strength alone, but not in combination with reduced respiratory muscle mass, may be sufficient for the identification of at‐risk individuals with reduced pulmonary function. The significant prognostic significance of FVC in our study population supports this consideration. In addition, the position paper for respiratory sarcopenia [29] adopted appendicular lean mass as a surrogate measure of respiratory muscle mass. However, given the lack of association between diaphragm thickness and appendicular lean mass, the diagnostic schema using appendicular lean mass may need further consideration. A positive association between lean body mass and respiratory muscle strength [31] also supports this consideration.

SAF‐AGE levels were inversely associated with diaphragm thickness. AGEs include several heterogeneous molecules that are non‐enzymatically generated by the glycation of proteins. Several studies have found an inverse association between the circulating levels of carboxymethyl lysine, a major AGE molecule, with grip strength [32] and gait speed [33] in older adults. In our previous study, we also observed an association between SAF‐AGE levels and low SMI and weak handgrip strength in the Nagahama study population [34], suggesting that AGE accumulation may have a harmful effect not only on appendicular skeletal muscle but also diaphragm. The deleterious effects of AGEs may be attributable to the formation of covalent cross‐links with proteins. Proteins that constitute the extracellular matrix including skeletal muscle are most susceptible to modification, which results in changes in skeletal muscle properties and decreases muscle strength [35]. Dietary intake of AGEs is a major determinant of circulating AGE levels [36]. In addition, AGEs levels may also reflect the long‐term glycaemic profile [37], and poor glycaemic profile is a risk factor for sarcopenia [38, 39] mostly in patients with Type 2 diabetes. However, the association between SAF‐AGEs and diaphragm thickness observed in this study was independent of insulin resistance, suggesting that SAF‐AGEs may themselves exert a deleterious effect on the diaphragm. A plausible reason for the lack of association between glycaemic level and diaphragm thickness was that the glycaemic levels in this study population were not high enough to adversely affect skeletal muscle. In our previous study, glycaemic levels in patients with diabetes but not in the general population were associated with low muscle mass [40].

A key strength of this study was large sample size with echography‐measured diaphragm thickness. To the best of our knowledge, no previous studies have reported the descriptive epidemiology of diaphragm thickness in a population comparable in size to this study. The longitudinal study setting was another strength, revealing the prognostic significance of diaphragm thickness. However, the study population was exclusively Japanese who have a smaller body size compared to the European population [41]. The findings may not be entirely generalizable to other populations. Similar studies in other ethnic populations may strengthen the present findings.

In conclusion, in this study, diaphragm thickness showed limited prognostic significance for all‐cause mortality. This was likely attributable to its differential association with common risk factors for sarcopenia compared to indices related to skeletal muscle of the extremities and the lack of direct association with appendicular skeletal muscle mass and muscle strength.

## Conflicts of Interest

The Department of Respiratory Care and Sleep Control Medicine at Kyoto University is funded by endowments from Philips‐Respironics, Fukuda Denshi, Fukuda Lifetec‐Keiji and Resmed to Kyoto University. The Department of Sleep Medicine and Respiratory Care, Division of Respiratory Medicine, Nihon University of Medicine is funded by endowments from Philips‐Respironics, Fukuda Denshi, Fukuda Lifetec‐Tokyo and Resmed to Nihon University. The authors declare no conflicts of interest.

## Supporting information


**Table S1.** Simple correlation coefficient between diaphragm thickness and age and anthropometric factors.
**Table S2.** Linear regression analysis for diaphragm thickness by sex.
**Table S3.** Causal mediation analysis between appendicular lean mass and diaphragm thickness in older adults (≥ 65 years of age).
**Table S4.** Linear regression analysis for spirometry parameters (*N* = 3135).
**Figure S1.** Age distribution of study participants.
**Figure S2.** Schematic image of the causal mediation analysis for the diaphragm thickness.
