# Supplementary material for: Descriptive Epidemiology and Prognostic Significance of Diaphragm Thickness in the General Population: The Nagahama Study
Source: J Cachexia Sarcopenia Muscle. 2025 Jan 25;16(1):e13690. doi: 10.1002/jcsm.13690 (PMC11761687; doi:10.1002/jcsm.13690)
Supplement: Supplementary file 1 — Table S1. Simple correlation coefficient between diaphragm thickness and age and anthropometric factors. Table S2. Linear regression analysis for diaphragm thickness by sex. Table S3. Causal mediation analysis between appendicular lean mass and diaphragm thickness in older adults (≥ 65 years of age). Table S4. Linear regression analysis for spirometry parameters (N = 3135). Figure S1. Age distribution of study participants. Figure S2. Schematic image of the causal mediation analysis for the diaphragm thickness. [file JCSM-16-e13690-s001.docx]

# Supplementary materials

Descriptive epidemiology of diaphragm thickness

in relation to skeletal muscle mass and pulmonary function

the Nagahama study

# Correspondence

Yasuharu Tabara

Graduate School of Public Health,

Shizuoka Graduate University of Public Health

Kita-ando 4-27-2, Aoi-ku, Shizuoka 420-0881, Japan

Tel: +81-54-295-5400, Fax: +81-54-248-3520

E-mail: tabara@s-sph.ac.jp

**Supplementary Table 1**. Simple correlation coefficient between diaphragm thickness and age and anthropometric factors

|  | Men | | | | |  | Women | | | | |
| --- | --- | --- | --- | --- | --- | --- | --- | --- | --- | --- | --- |
|  | Diaphragm thickness | | | | |  | Diaphragm thickness | | | | |
|  | at end-expiration | |  | at end-inspiration | |  | at end-expiration | |  | at end-inspiration | |
|  | r | P |  | r | P |  | r | P |  | r | P |
| Age, years | −0.003 | 0.917 |  | −0.079 | 0.012 |  | 0.111 | <0.001 |  | 0.085 | <0.001 |
| Body height, cm | 0.019 | 0.554 |  | 0.044 | 0.159 |  | −0.051 | 0.015 |  | −0.055 | 0.008 |
| Body weight, kg | 0.179 | <0.001 |  | 0.112 | <0.001 |  | 0.219 | <0.001 |  | 0.094 | <0.001 |
| BMI, kg/m^2^ | 0.199 | <0.001 |  | 0.108 | 0.001 |  | 0.263 | <0.001 |  | 0.130 | <0.001 |
| Waist circumference, cm | 0.215 | <0.001 |  | 0.103 | 0.001 |  | 0.259 | <0.001 |  | 0.117 | <0.001 |
| SMI, kg/m^2^ | 0.157 | <0.001 |  | 0.124 | <0.001 |  | 0.182 | <0.001 |  | 0.104 | <0.001 |

Values are simple correlation coefficient.

**Supplementary Table 2**. Linear regression analysis for diaphragm thickness by sex

|  |  | Diaphragm thickness | | | | |  | Diaphragm  thickening fraction | |
| --- | --- | --- | --- | --- | --- | --- | --- | --- | --- |
|  |  | at end-expiration | |  | at end-inspiration | |  |  |  |
|  |  | β | P |  | β | P |  | β | P |
| Men | Age, years | −0.032 | 0.383 |  | −0.075 | 0.042 |  | −0.070 | 0.055 |
|  | Waist circumference, cm | 0.249 | <0.001 |  | 0.120 | 0.001 |  | −0.150 | <0.001 |
|  | Smoking habit | −0.027 | 0.386 |  | −0.032 | 0.308 |  | −0.027 | 0.383 |
|  | Handgrip strength, kg | −0.036 | 0.316 |  | 0.038 | 0.299 |  | 0.070 | 0.051 |
|  | HOMA-IR, log-normalized | −0.044 | 0.218 |  | −0.029 | 0.421 |  | 0.006 | 0.861 |
|  | Exercise habit | 0.069 | 0.032 |  | 0.076 | 0.019 |  | 0.004 | 0.900 |
| Women | Age, years | 0.051 | 0.036 |  | 0.084 | 0.001 |  | 0.042 | 0.094 |
|  | Waist circumference, cm | 0.261 | <0.001 |  | 0.123 | <0.001 |  | −0.153 | <0.001 |
|  | Smoking habit | 0.007 | 0.752 |  | 0.042 | 0.048 |  | 0.042 | 0.048 |
|  | Handgrip strength, kg | 0.033 | 0.134 |  | 0.036 | 0.104 |  | 0.000 | 0.991 |
|  | HOMA-IR, log-normalized | −0.025 | 0.294 |  | −0.047 | 0.056 |  | −0.043 | 0.074 |
|  | Exercise habit | 0.041 | 0.052 |  | 0.001 | 0.960 |  | −0.045 | 0.039 |

Smoking habit includes current and past smoking. β: standardized regression coefficient, VIF: variance inflation factor, HOMA-IR: homeostasis model assessment of insulin resistance.

**Supplementary Table 3**. Causal mediation analysis between appendicular lean mass and diaphragm thickness in olde adults (≥65 years of age)

|  | Appendicular  lean mass | Diaphragm thickness | | | | | | | |
| --- | --- | --- | --- | --- | --- | --- | --- | --- | --- |
|  |  | at end-expiration | | |  | | at end-inspiration | | |
|  |  | coefficient | s.e. | P |  | coefficient | | s.e. | P |
| Natural indirect effect | Q1 |  | reference |  |  |  | | reference |  |
| via waist circumference | Q2 | 0.025 | 0.012 | 0.040 |  | 0.016 | | 0.019 | 0.403 |
|  | Q3 | 0.104 | 0.024 | <0.001 |  | 0.086 | | 0.050 | 0.085 |
|  | Q4 | 0.191 | 0.038 | <0.001 |  | 0.186 | | 0.063 | 0.003 |
| Natural direct effect | Q1 |  |  |  |  |  | |  |  |
|  | Q2 | -0.005 | 0.041 | 0.897 |  | 0.055 | | 0.074 | 0.458 |
|  | Q3 | 0.057 | 0.046 | 0.215 |  | 0.159 | | 0.093 | 0.088 |
|  | Q4 | 0.028 | 0.052 | 0.598 |  | 0.029 | | 0.097 | 0.768 |
| Total effect | Q1 |  |  |  |  |  | |  |  |
|  | Q2 | 0.020 | 0.041 | 0.625 |  | 0.071 | | 0.072 | 0.325 |
|  | Q3 | 0.161 | 0.046 | <0.001 |  | 0.244 | | 0.080 | 0.002 |
|  | Q4 | 0.219 | 0.050 | <0.001 |  | 0.215 | | 0.084 | 0.011 |

Adjusted factors were age, sex, smoking habit, homeostasis model assessment of insulin resistance (log normalized), grip strength, and exercise habit. Quartiles of appendicular lean mass were calculated separately for men and women and then combined to avoid potential sex-based differences. s.e. indicates standardized error.

**Supplementary Table 4**. Linear regression analysis for spirometry parameters (N = 3,135)

|  |  | FVC | |  | FEV_1_ | |  | FEV_1_/FVC | |
| --- | --- | --- | --- | --- | --- | --- | --- | --- | --- |
|  |  | β | P |  | β | P |  | β | P |
| Model 1 | Age, years old | −0.341 | <0.001 |  | −0.467 | <0.001 |  | −0.433 | <0.001 |
|  | Sex, men | 0.385 | <0.001 |  | 0.387 | <0.001 |  | 0.058 | 0.036 |
|  | Body height, cm | 0.427 | <0.001 |  | 0.338 | <0.001 |  | −0.220 | <0.001 |
|  | Body weight, kg | 0.011 | 0.428 |  | 0.025 | 0.069 |  | 0.042 | 0.058 |
|  | Smoking habit | −0.039 | 0.001 |  | −0.071 | <0.001 |  | −0.108 | <0.001 |
|  | Diaphragm thickness at end-expiration, cm | −0.010 | 0.343 |  | −0.004 | 0.715 |  | 0.017 | 0.313 |
| Model 2 | Age, years old | −0.342 | <0.001 |  | −0.468 | <0.001 |  | −0.432 | <0.001 |
|  | Sex, men | 0.377 | <0.001 |  | 0.380 | <0.001 |  | 0.059 | 0.033 |
|  | Body height, cm | 0.432 | <0.001 |  | 0.341 | <0.001 |  | −0.222 | <0.001 |
|  | Body weight, kg | 0.002 | 0.860 |  | 0.018 | 0.171 |  | 0.046 | 0.033 |
|  | Smoking habit | −0.039 | 0.001 |  | −0.071 | <0.001 |  | −0.108 | <0.001 |
|  | Diaphragm thickness at end-inspiration, cm | 0.032 | 0.002 |  | 0.032 | 0.002 |  | 0.007 | 0.697 |
| Model 3 | Age, years old | −0.339 | <0.001 |  | −0.465 | <0.001 |  | −0.432 | <0.001 |
|  | Sex, men | 0.381 | <0.001 |  | 0.384 | <0.001 |  | 0.061 | 0.027 |
|  | Body height, cm | 0.426 | <0.001 |  | 0.336 | <0.001 |  | −0.222 | <0.001 |
|  | Body weight, kg | 0.015 | 0.237 |  | 0.031 | 0.021 |  | 0.046 | 0.034 |
|  | Smoking habit | −0.039 | 0.001 |  | −0.071 | <0.001 |  | −0.108 | <0.001 |
|  | Diaphragm Thickening fraction, % | 0.042 | <0.001 |  | 0.038 | <0.001 |  | −0.007 | 0.663 |

Analysis was performed in a population without taking inhaled medication and without having histories of pulmonary disease (N = 3,135). Smoking habit includes current and past smokers. FVC: forced vital capacity, FEV_1_: forced expiratory volume in 1 sec, β: standardized regression coefficient.

**Supplementary Figure 1**. Age distribution of study participants

**Supplementary Figure 2**. Schematic image of the causal mediation analysis for the diaphragm thickness

In addition to the three factors shown in the schema, possible confounding factors including age, sex, homeostasis model assessment of insulin resistance (log normalized), grip strength, and exercise habit were further adjusted.
